# Comparing DNA metabarcoding with light microscopy to identify eukaryotic phytoplankton in the Baltic Sea, Kattegat and Skagerrak

**DOI:** 10.1038/s41598-026-48838-z

**Published:** 2026-05-19

**Authors:** Anders Torstensson, Sonia Brugel, Anders F. Andersson, Mikael Hedblom, Krzysztof T. Jurdzinski, Bengt Karlson, Meike A. C. Latz, Markus Lindh, Jenny Lycken, Agneta Andersson

**Affiliations:** 1 Swedish Meteorological and Hydrological Institute, Västra Frölunda, Sweden; 2 Department of Ecology, Environment and Geoscience, Umeå University, 901 87 Umeå, Sweden; 3 Umeå Marine Sciences Centre, 905 71 Hörnefors, Sweden; 4 Department of Gene Technology, SciLifeLab, KTH Royal Institute of Technology, Stockholm, Sweden; 5 Department of Plant and Environmental Sciences, University of Copenhagen, Frederiksberg C, Denmark

**Keywords:** Ecology, Ecology, Microbiology, Ocean sciences

## Abstract

Marine phytoplankton monitoring has long relied on microscopy, but DNA metabarcoding has recently emerged as a complementary approach. This study assessed the applicability of DNA metabarcoding of the 18S ribosomal RNA gene in marine monitoring and compared its results with conventional microscopy. We analyzed data from 232 surface water samples from 17 monitoring stations in the Baltic Sea, Kattegat, and Skagerrak. Metabarcoding detected more orders, genera, and species than microscopy, with a 43% overlap in the most common genera identified by both methods. Despite attempts to normalize sequence reads to spike-in DNA or DNA concentrations, the correlations between abundances derived from the two methods were weak, though varied considerably between taxonomic groups and geographical areas. Correlations were consistently stronger when using carbon and biovolume concentrations than cell abundances. Our results highlight the potential of metabarcoding to expand biodiversity assessments and advance our understanding of microbial biodiversity in marine ecosystems. As a complement to microscopy, it can enhance existing monitoring efforts. Future improvements in reference database completeness, adoption of long-read sequencing technologies, and better characterization of gene copy number variability per cell are needed to further extend the applicability of metabarcoding for quantitative analyses.

**Supplementary Information:**

The online version contains supplementary material available at 10.1038/s41598-026-48838-z.

## Introduction

Monitoring phytoplankton communities is important for understanding ecosystem health, assessing water quality, and studying the impacts of environmental changes. Accurately identifying and quantifying the taxa present in an ecosystem are crucial to ecosystem monitoring programs^1^. Conventional methods in phytoplankton community analysis are performed by trained taxonomists who identify taxa based on morphological characters in preserved samples using light microscopy [e.g^2,3^.. These monitoring techniques have been instrumental in advancing our understanding of phytoplankton dynamics. However, they often require significant time, labor, and extensive taxonomic expertise. Small taxa are particularly difficult or even impossible to identify due to technical limitations of light microscopy, which in combination with the small amount of volume that is generally analyzed, limits the assessment of protist biodiversity^4^. Inconsistency between taxonomists^5^ also presents a challenge for long-term monitoring programs. The conventional methods have additional limitations, such as challenges associated with species identification and enumeration in preserved samples^6–8^.

Advancements in technology and the development of automated systems are helping to improve the efficiency and accuracy of phytoplankton monitoring, such as molecular approaches^9–12^ and automated image analysis^9,13^, enabling more comprehensive assessments of these vital organisms in aquatic ecosystems. The use of molecular methods, such as DNA metabarcoding, for studying phytoplankton biodiversity has developed rapidly during the first two decades of the 21th century^4^. Metabarcoding provides a tool for describing microbial biodiversity and community composition of large sample numbers for a relatively small cost compared to the labor-intense microscopy analysis^14^. By sequencing specific DNA marker genes (e.g. the 18S ribosomal RNA (rRNA) gene for eukaryotic organisms), information on the taxa present in an ecosystem can be gained on a sequence-level, thereby enabling the distinction between taxa that are impossible to distinguish by light-microscopy. Several studies have compared morphological methods to metabarcoding in aquatic eukaryotes^11,14–21^, but few have focused on brackish environments^22^. To our knowledge, no previous studies have compared metabarcoding and microscopy across such a wide salinity range in marine-brackish waters supporting the diverse phytoplankton communities of the Baltic Sea, Kattegat, and Skagerrak.

The DNA sequences obtained from metabarcoding are taxonomically classified by comparisons with a database of reference sequences. Incomplete coverage of taxa in the reference databases therefore poses a limitation of the method. The issue is likely to diminish over time as more organisms are analyzed and more sequences become available in curated resources such as the Protist Reference database^23^. Moreover, the taxonomic resolution of any single marker gene varies across taxa, and a given marker may lack sufficient discriminatory power for certain groups^4,24^. Consequently, selecting appropriate, high-resolution markers, and using multiple markers when necessary, is essential to achieve reliable taxonomic assignment of taxa of particular interest^25,26^. An additional limitation arises from the variable quality of public reference databases, which may contain incorrectly annotated or unreferenced sequences, as well as unresolved taxonomic conflicts that can propagate errors in downstream analyses^27^. Furthermore, the relatively short sequence usually analyzed with short-read sequencing technologies such as Illumina limits the ability to distinguish between related taxa. As a result of those limitations, not all taxa can currently be correctly assigned to species or even genus level, which for instance can be problematic in terms of identifying specific taxa that are responsible for harmful algal blooms (HABs)^16^. As reference databases evolve, the coverage of taxa significantly improves, enabling the re-annotation of previously collected data, creating an advantage for long-term monitoring programs. Next to these methodological limitations, it is currently still a challenge to accurately infer absolute abundance from metabarcoding, primarily due to substantial variation in gene copy numbers per cell across taxa^28,29^. This information is crucial for modeling and water quality assessment purposes. Nevertheless, studies have indicated that the incorporation of spike-in DNA as an internal standard for DNA sequence abundance could offer a potential solution^30–32^, yet, to our knowledge, the method remains untested in aquatic environments. The implementation of metabarcoding in marine monitoring will broaden our understanding of microbial biodiversity, and will act to complement conventional methods of monitoring phytoplankton communities, but face several challenges in terms of method harmonization between nations sharing marine areas^26^.

**Fig. 1:**
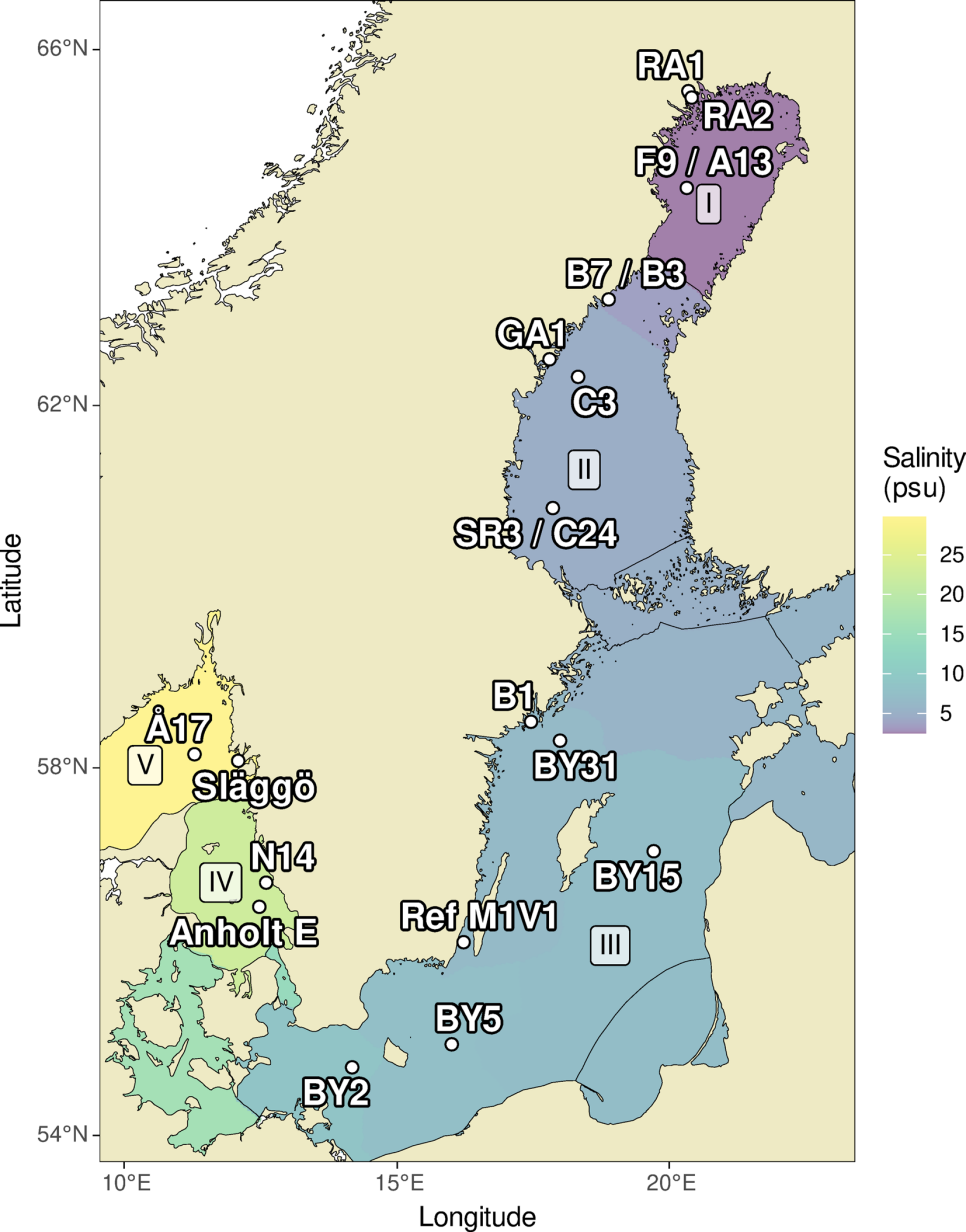
Map showing sampling locations. Average surface (≤ 10 m depth) salinities between 1980–2023 are displayed for the sub-basins defined by the Helsinki Commission and the Oslo and Paris Conventions (data originating from the Swedish National Oceanographic Data Centre at the Swedish Meteorological and Hydrological Institute, and are available in https://shark.smhi.se/en/). The stations are grouped by the five major sub-basins; I: Bothnian Bay, II: Bothnian Sea, III: Baltic Proper, IV: Kattegat and V: Skagerrak. Full station names are listed in Table 1. The map was created using the R library ‘ggOceanMaps’^51,53^.

The Baltic Sea, Kattegat, and Skagerrak system exhibits a pronounced surface salinity gradient, spanning roughly from 2 in the Bothnian Bay to about 32 in the Skagerrak (Fig. 1 and Table 1). This unique system hosts a diverse array of phytoplankton taxa, encompassing both freshwater and marine species^10,33^. The identification of phytoplankton based on morphological traits within this system requires extensive taxonomic expertise, underscoring the advantages offered by molecular methods in expanding biodiversity assessment. In this study, phytoplankton community analyses using the Utermöhl method were compared with DNA metabarcoding in an ecosystem characterized by a wide range of phytoplankton taxa. Between January 2019 and February 2020, a total of 232 surface samples were collected from 17 monitoring stations in the Baltic Sea, Kattegat and Skagerrak (Fig. 1, Table 1), as part of the Swedish National Marine Monitoring Program. In addition to the standard sampling program^34,35^ that includes phytoplankton analysis by light microscopy, 18S rRNA gene metabarcoding was analyzed. The datasets^36^ were used to investigate disparities between the two methods and to assess the applicability of metabarcoding in marine monitoring.

**Table 1:** Summary of sampled stations during the study (sorted by salinity). The proportion of phytoplankton refers to the percentage of 18S rRNA reads that were annotated to the selected classes (after removal of spike and metazoan sequences) in Table 2.

Station name	Full station name	Sea basin	*n* samples	*n* sampling months	Sampling depth (m)	Salinity	Proportion phytoplankton (%)
RA1	RÅNEÅ−1	Bothnian Bay	6	6	0–5	2.1	38
RA2	RÅNEÅ−2	Bothnian Bay	12	11	0–10	2.3	43
F9/A13	F9/A13	Bothnian Bay	11	10	0–10	3.0	50
C3	C3	Bothnian Sea	12	10	0–10	5.1	62
GA1	GAVIK-1	Bothnian Sea	12	10	0–10	5.1	61
B3/B7	B3/B7	Bothnian Sea	22	12	0–10	4.3	59
SR3/C24	SR3/C24	Bothnian Sea	3	3	0–10	5.3	57
Ref M1V1	REF M1V1	Baltic Proper	10	10	0–10	7.1	56
BY15	BY15 GOTLANDSDJ	Baltic Proper	13	11	0–10	7.2	68
BY2	BY2 ARKONA	Baltic Proper	13	11	0–10	8.1	53
B1	B1	Baltic Proper	23	12	0–20	6.4	58
BY31	BY31 LANDSORTSDJ	Baltic Proper	20	11	0–20	6.5	66
BY5	BY5 BORNHOLMSDJ	Baltic Proper	13	11	0–10	7.7	53
Anholt E	ANHOLT E	Kattegat	20	11	0–10	21.3	66
N14	N14 FALKENBERG	Kattegat	12	11	0–10	22.5	67
Släggö	SLÄGGÖ	Skagerrak	18	11	0–10	26.6	64
Å17	Å17	Skagerrak	12	10	0–10	31.7	56

## Results

### Influence of sample volume on alpha diversity

**Fig. 2:**
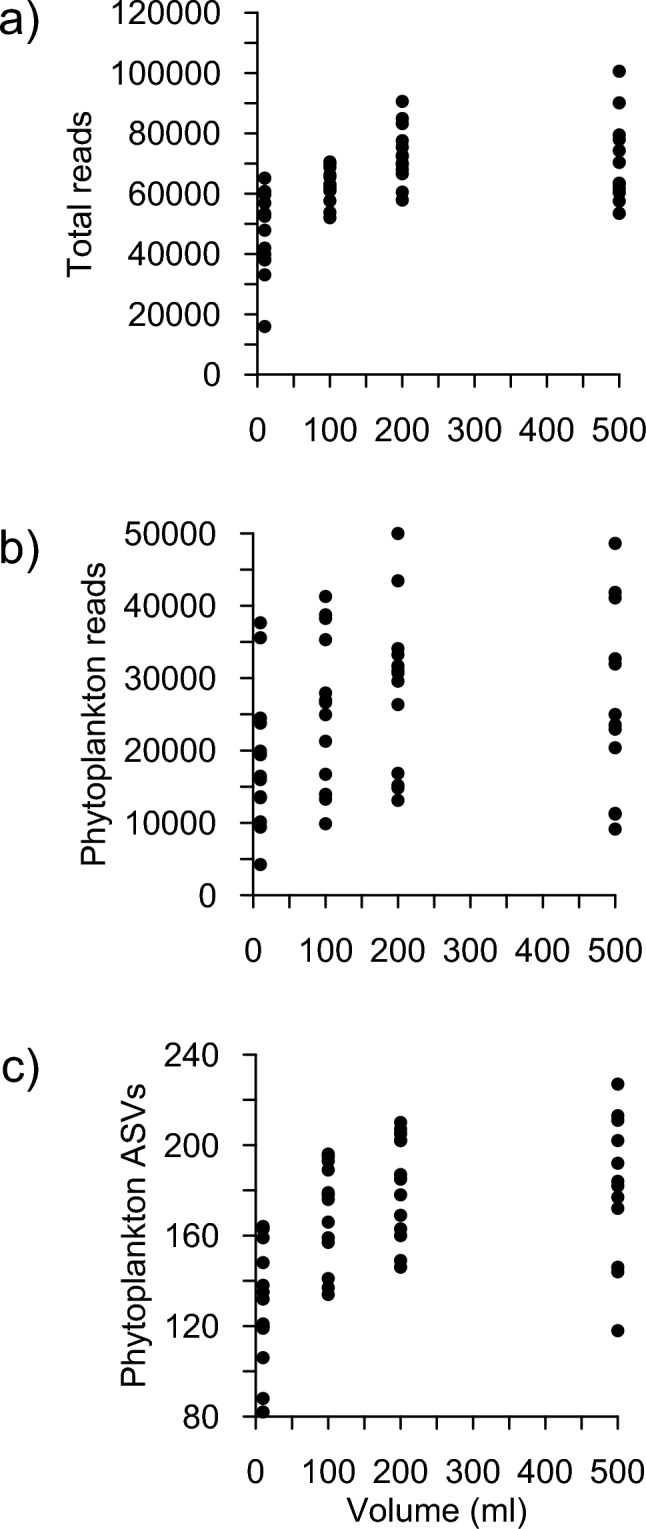
Number of total reads (**a**), number of phytoplankton reads (**b**) and number of phytoplankton ASVs (**c**) for the different filtered volumes tested (10, 100, 200 and 500 ml).

The number of total reads, phytoplankton reads and phytoplankton ASVs increased with the sample volume filtered until 200 ml (Fig. 2); however, there was no difference in the number of phytoplankton ASVs detected between 200 and 500 ml (Mann–Whitney U test, *p* = 1). Using metabarcoding, most of the phytoplankton diversity was already described with 200 ml of water sample.

### Taxon detection and biogeographic overlap: metabarcoding and microscopy comparison

In this study, a total of 266 distinct taxa, which are part of the defined eukaryotic phytoplankton community (Table 2), were identified using the Utermöhl method. A total of 2,346 unique ASVs could be assigned to the classes that were used for defining the eukaryotic phytoplankton community (Table 2). ASV lengths ranged from 265 to 490 bp (mean = 378 bp).

**Table 2:** Selected phytoplankton classes that defines the eukaryotic micro- and nanophytoplankton community in this study and was identified both by 18S rRNA metabarcoding and the Utermöhl method. The taxonomy follows the classifications and structure defined in the current AlgaeBase taxonomical database^37^ database, with the exception from Coscinodiscophyceae and Mediophyceae that were lumped together for a better comparison to microscopy, where unknown centric diatom cells are often reported to the order Centrales.

Supergroup	Division	Subdivision	Class
TSAR	Stramenopiles	Gyrista	Bacillariophyceae
TSAR	Stramenopiles	Gyrista	Coscinodiscophyceae + Mediophyceae
Archaeplastida	Chlorophyta		Chlorodendrophyceae
Archaeplastida	Chlorophyta		Chlorophyceae
TSAR	Stramenopiles	Gyrista	Chrysophyceae
Haptista	Haptophyta		Coccolithophyceae
Cryptista	Cryptophyta		Cryptophyceae
TSAR	Stramenopiles	Gyrista	Dictyochophyceae
TSAR	Alveolata	Dinoflagellata	Dinophyceae
TSAR	Stramenopiles	Gyrista	Eustigmatophyceae
TSAR	Alveolata	Dinoflagellata	Noctilucophyceae
Archaeplastida	Chlorophyta		Pyramimonadophyceae
TSAR	Stramenopiles	Gyrista	Raphidophyceae
Archaeplastida	Chlorophyta		Trebouxiophyceae

**Fig. 3:**
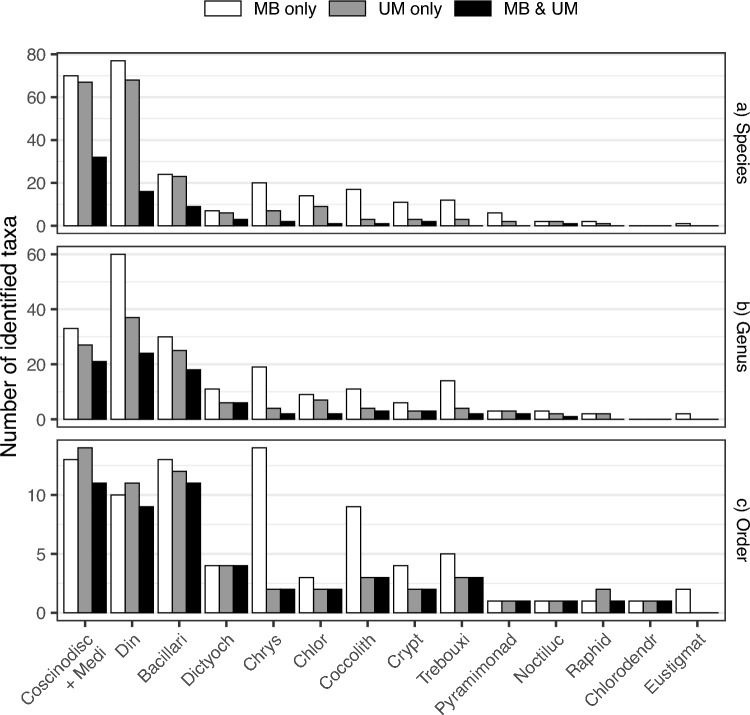
Comparison of taxonomic identification using the Utermöhl method (UM) and metabarcoding (MB) for 14 selected phytoplankton classes (ophyceae-truncated). The figure depicts the number of unique and shared taxa at species, genus, and order levels.

After aligning with the current AlgaeBase taxonomic database^37^ and refining the taxonomy table derived from PR^2^ (Supplementary tables S1 and S2), the metabarcoding method resulted in a larger number of identified orders, genera, and species compared to the Utermöhl method. Of the detected orders, 81 were unique to metabarcoding, 58 to Utermöhl, and 51 were shared. At the genus level, 203 were unique to metabarcoding, 124 to Utermöhl, and 84 were shared, while at the species level the corresponding numbers were 263, 194, and 67 (Fig. 3). While metabarcoding and the Utermöhl method yielded a similar number of identified species for the two most abundant classes, Coscinodiscophycae + Mediophyceae and Dinophyceae, only 46% and 24% of the species, respectively, were shared between the two methods (Fig. 3a).

**Fig. 4:**
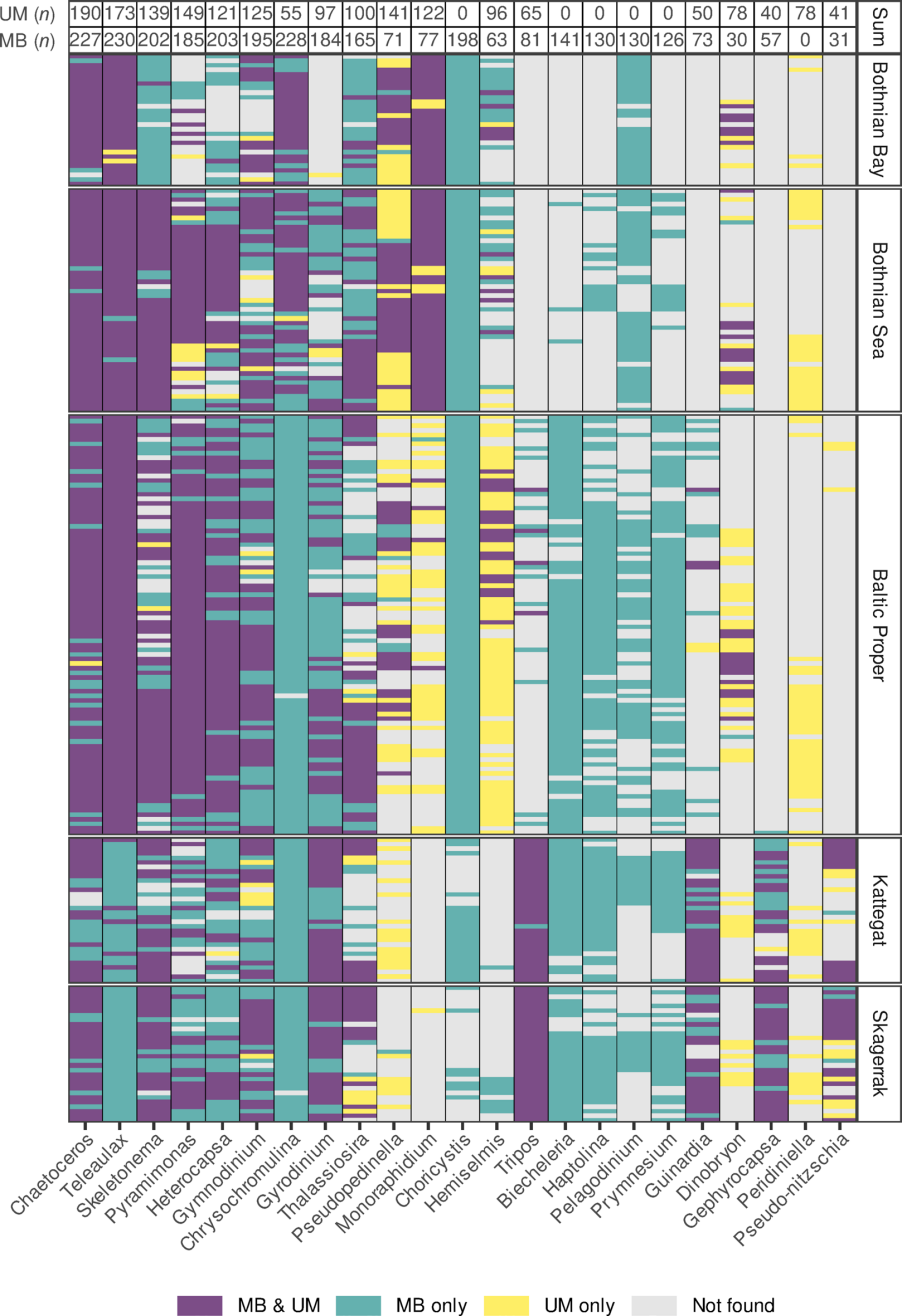
Comparison of the presence of the 23 most abundant genera using 18S rRNA gene metabarcoding (MB) and the Utermöhl method (UM). The number of total observations by each method is shown at the top of the figure. Not found indicates that the genus was not detected by either method in the respective sub-basin.

To compare the most abundant genera across datasets, the 15 highest-ranked genera (based on relative abundance) were identified independently for each method. Combining these two lists, which partially overlapped, resulted in a total of 23 unique genera. Among these 23 genera, the following were found exclusively in the metabarcoding dataset: *Choricystis* (Trebouxiophyceae), *Biecheleria* (Dinophyceae), *Haptolina* (Coccolithophyceae), *Pelagodinium* (Dinophyceae), and *Prymnesium* (Coccolithophyceae), while *Peridiniella* (Dinophyceae) was solely present in the dataset generated by the Utermöhl method (Fig. 4). There was a 43% overlap (1474 of a total of 3413 observations) between the two methods in terms of presence observations for the 23 most abundant genera. Additionally, metabarcoding identified 1,553 instances of presence that went undetected by the Utermöhl method (Fig. 4), whereas the Utermöhl method recorded 386 instances of presence that were not detected by metabarcoding (Fig. 4). Remarkably, uneven patterns of detection were observed for some genera, which were detected by microscopy and not (or very sparsely) by metabarcoding only in some specific basins, as for example, *Monoraphidium* and *Hemiselmis* in the Baltic Proper, or *Pseudopedinella* and *Dinobryon* in the Kattegat-Skagerrak (Fig. 4).

Rare taxa were defined as taxa with a relative abundance < 0.01% in all samples. In the metabarcoding dataset 215 ASVs were rare, among which 24 ASVs could be annotated to 20 species, however only 4 species had a cumulative abundance < 0.01%. The rare species, *Protodinium simplex* (Dinophyceae), *Tetraedron minimum* (Chlorophyceae), *Pyramimonas parkae* (Pyramimonadophyceae) and *Yihiella yeosuensis* (Dinophyceae), were only found by metabarcoding. In the microscopy dataset, six species were rare: *Gyrodinium fusiforme* (Dinophyceae), *Striatella unipunctata* (Bacillariophyceae), *Odontella aurita* (Mediophyceae), *Gymnodinium stellatum* (Dinophyceae), *Thalassiosira eccentrica* (Mediophyceae) and *Tabellaria fenestra* (Bacillariophyceae), the three latest species were exclusively found by microscopy.

### Eukaryotic phytoplankton community composition

**Fig. 5:**
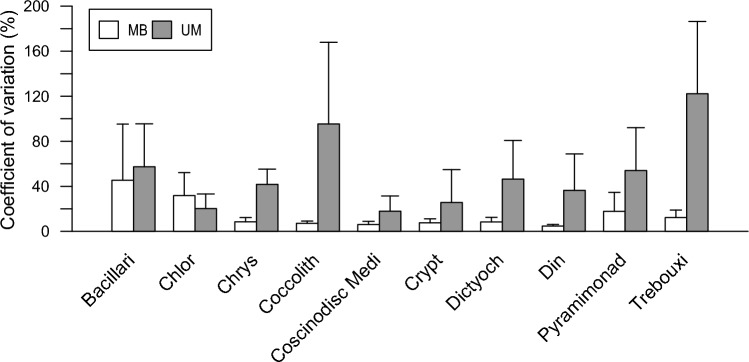
Coefficient of variation of the relative proportion of 10 selected classes (ophyceae-truncated) derived from partial 18S rRNA gene sequence reads (MB) and microscopic cell abundance (UM), issued from method replication tests (six tests of five replicates). Error bars denotes standard deviation.

The reproducibility of the metabarcoding and Utermöhl methods was assessed by analyzing five replicate samples from six sampling occasions (two stations sampled at three time-points each). The assessment was based only on the 10 phytoplankton classes present in these samples. Metabarcoding gave more reproducible relative abundance estimates of these classes, as shown by the significantly lower coefficient of variation (Mann-Withney U-test, p < 0.05) for Chrysophyceae, Coccolithophyceae, Dictyohcophyceae, Dinophyceae and Trebouxiophyceae compared to microscopy (Fig. 5).

**Fig. 6:**
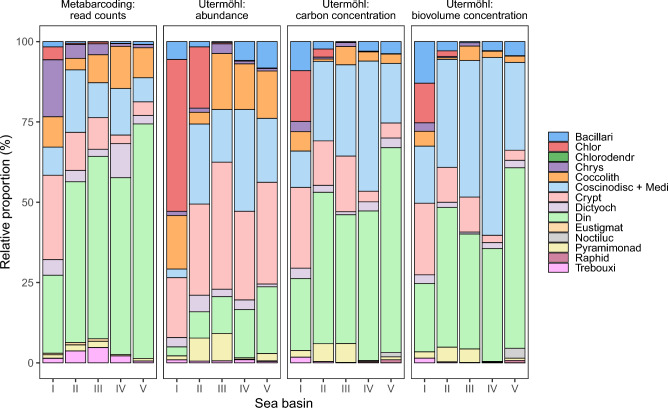
Average relative proportion of 14 selected classes (ophyceae-truncated) to the defined eukaryotic phytoplankton community (Table 2), derived from partial 18S rRNA gene sequence reads, microscopic cell abundance, carbon and biovolume concentration, as determined by microscopic analysis. The stations are categorized into five major sub-basins; I: Bothnian Bay, II: Bothnian Sea, III: Baltic Proper, IV: Kattegat and V: Skagerrak, according to Fig. 1.

The eukaryotic phytoplankton community composition was evaluated using four different relative measures based on read counts from metabarcoding, cell abundance from the Utermöhl method, and carbon and biovolume concentrations from the Utermöhl method. Across all sea basins, the dominant class in the metabarcoding dataset was Dinophyceae (51.8%), followed by Coscinodiscophyceae + Mediophyceae (12.2%) (Fig. 6). In contrast, based on cell abundance from the Utermöhl method, the community was primarily dominated by Cryptophyceae (29.2%), followed by Coscinodiscophyceae + Mediophyceae (19.1%). Dinophyceae accounted for only 11.6% of the total eukaryotic phytoplankton community. However, when considering carbon and biovolume concentrations, Dinophyceae were the dominant group, comprising 44.0% and 38.4%, respectively, followed by Coscinodiscophyceae + Mediophyceae at 24.7% and 35.3%, respectively.

**Fig. 7:**
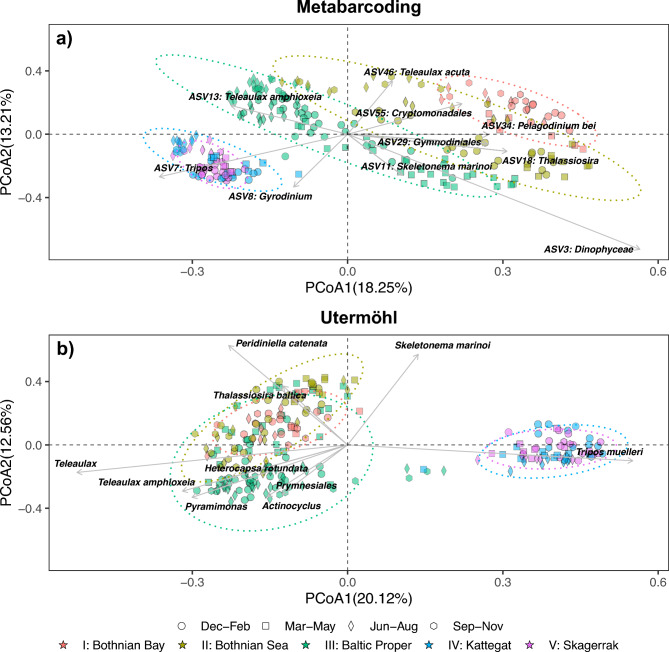
Principal coordinates analysis (PCoA) of the eukaryotic phytoplankton community, showcasing the Bray–Curtis dissimilarity in the 18S rRNA gene metabarcoding dataset (**a**) and carbon concentration derived from the Utermöhl method (**b**). The arrows depict the top 10 taxa contributing to the observed dissimilarity between samples.

Principal Coordinates Analysis (PCoA) using Bray–Curtis dissimilarity and PERMANOVA demonstrated distinct differences between phytoplankton communities in various sea basins, both in the metabarcoding dataset (*p* < 0.001, *F*_4,227_ = 16.2 PERMANOVA) and the Utermöhl method dataset (*p* < 0.001, *F*_4,227_ = 15.7, PERMANOVA), particularly in separating samples from the Baltic Sea and the Kattegat-Skagerrak (Fig. 7). Pairwise PERMANOVA analysis indicated significant differences in community composition between all sea basins in the metabarcoding and the Utermöhl method using carbon concentration dataset (*p* < 0.001, PERMANOVA). The Utermöhl method could, however, not discriminated between Skagerrak and Kattegat based on abundance data (*p* = 0.123, PERMANOVA), although analysis of multivariate homogeneity of group dispersions indicated heterogenous variances in both datasets (*p* < 0.01 PERMDISP).

PCoA plots provided insights into the specific ASVs or taxa responsible for the dissimilarities observed between samples. In the metabarcoding dataset, ASVs associated with the genera *Tripos* and *Gyrodinium* were the key contributors to distinguishing Kattegat-Skagerrak from the Baltic Sea samples. However, in the microscopy dataset, the dinoflagellate species *Tripos muelleri* was the primary taxa contributing to the dissimilarity between Kattegat-Skagerrak and the Baltic Sea (Fig. 7), while the cryptophyte genera *Teleaulax* and the species *Teleaulax amphioxeia* were specifically associated with samples from the Baltic Sea. Interestingly, *Teleaulax* was detected in Kattegat-Skagerrak according to the metabarcoding results (Fig. 4). However, morphologic features enabling the taxonomic assignment of cells to the genus *Teleaulax* could be absent in the Kattegat-Skagerrak samples, resulting in the cells to be classified at the order level, i.e. Cryptomonadales.

### Evaluation of sequence read abundance normalization

In an attempt to derive absolute abundances from the metabarcoding sequence read abundances, different normalization techniques applied to the metabarcoding data were tested against quantifications obtained from the Utermöhl method: relative read abundance, spike-normalized read abundance and DNA concentration-normalized read abundance.

**Fig. 8:**
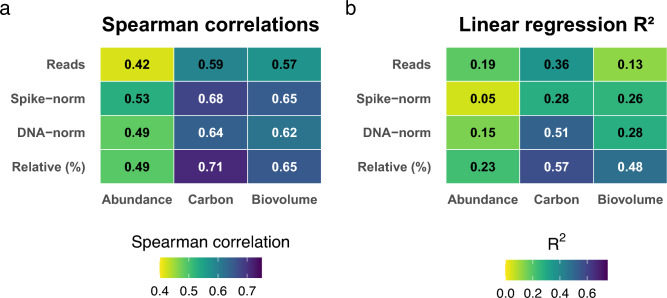
Spearman’s rank correlation coefficient (**a**) and R2-values (**b**) from linear regressions are presented to compare normalization techniques of 18S rRNA gene metabarcoding with the Utermöhl method. The analysis uses data that have been aggregated to class level. Each observation represents the abundance of a single class in a single sample, and all such class by sample combinations are included together when calculating each correlation or regression. All variables were log transformed before analysis, covering metabarcoding measures (read counts, spike-normalized counts, DNA-normalized counts, relative read abundance) and microscopy measures (cell abundance, carbon concentration, biovolume concentration).

Comparing the different normalization techniques on class level alone, spike-normalization generally improved the R^2^ fit and Spearman’s rank correlation coefficient relative to raw read abundances across all samples (Fig. 8). Raw read counts are shown as a baseline to illustrate the uncorrected sequencing signal, allowing the effect of normalization methods to be directly assessed. However, closer inspection of the R^2^ and rank-based correlation values revealed that spike normalization produced correlation coefficients similar to those obtained with relative normalization (Fig. 8a), but lower R^2^ values (Fig. 8b). Similarly, DNA normalization improved the fit compared with raw read counts and cell abundance, yet the resulting R^2^ and correlation values were comparable to, or lower than, those observed for relative read abundances (Fig. 8).

Carbon and biovolume concentrations were generally more strongly correlated with metabarcoding results than cell abundances, as indicated by higher R^2^ values and Spearman’s correlation coefficients, regardless of the normalization method used (Fig. 8). Comparing relative read abundances to relative carbon concentrations, rather than relative cell abundances, substantially improved both the linear fit and the correlation coefficient. While relative biovolume showed a stronger relationship than cell abundance, its correlation with metabarcoding results remained lower than that observed for carbon concentration.

There were also spatial differences in the success of spike-normalization. Cell abundances showed a stronger relationship in the Bothnian Bay, with an average adjusted R^2^ value of 0.62 (compared to 0.24 in the overall dataset, data not shown) for all classes with observations in both datasets (Fig. 8). The classes Coscinodiscophyceae + Mediophyceae, Coccolithophyceae, Cryptophyceae, Dictyochophyceae, Dinophyceae, and Pyramimonadophyceae displayed adjusted R^2^ values > 0.65 in this particular basin.

Normalization approaches had variable effects at the genus level. Overall, comparisons based on relative abundances yielded the highest R^2^ values for the most common taxa in the dataset (Supplementary Figure S3). However, spike-normalization improved correlations for certain genera, such as *Chrysochromulina*, whereas DNA-normalization resulted in higher R^2^ values for others, including *Gephyrocapsa* and *Thalassiosira*. Across methods, regressions against carbon concentration and biovolume generally revealed stronger relationships between metabarcoding and microscopy for most abundant genera compared to regressions based on cell abundances.

## Discussion

Metabarcoding has been proposed as a valuable addition to conventional microscopic methods for identifying cyanobacteria^38^, zooplankton^11,17,20^, diatom epiphyton^15^ and eukaryotic phytoplankton^11,14,16,18,19^. However, no previous studies, to the best of our knowledge, have explored the application of metabarcoding in a marine-brackish environment with such a wide range of salinity (approximately 2 to 32), which harbors a diverse array of phytoplankton communities. In our study, metabarcoding of the 18S rRNA gene provided several advantages over the conventional Utermöhl method, including lower costs. It detected a greater number of taxa and exhibited lower variation in relative abundance for most phytoplankton classes. Moreover, metabarcoding captured more effectively beta diversity compared to the Utermöhl method. The observed significant difference between Kattegat and Skagerrak from the PERMANOVA is likely a result of differences in statistical dispersion, rather than a true ecological distinction. Nonetheless, accurately normalizing sequence reads to precisely reflect cell abundance, biovolume, or carbon concentration, as determined by microscopic analysis, proved challenging.

### Biogeographic patterns revealed by metabarcoding and microscopy

While the number of identified species was comparable between the major classes Coscinodiscophyceae + Mediophyceae and Dinophyceae, the overlap in species names was relatively low. This discrepancy could partly be attributed to the relatively short sequence lengths (265–490 bp) obtained through metabarcoding of the 18S V4 region, which limit the ability to accurately assign species annotations due to identical DNA regions among closely related species^24^. The use of longer 18S rRNA gene sequences may help resolve some species-level discrepancies^25,39^, but will not address all issues.

The identification of commonly occurring genera was geographically distinct between both methods (Fig. 4). In some basins, a number of genera was only detected by microscopy, while in other basins these were found in both microscopy and metabarcoding datasets. The success of sequence annotation relies on the reference databases, which need to cover not only an extensive number of genera and species but also an extensive number of specimens from diverse environments for a given genus or species. The reference database (PR^2^ version 5.1.0) contains only 8 reference sequences for *Pseudopedinella.* The low number of references likely explains the absence of this genus in the metabarcoding dataset in the Skagerrak and Kattegat basins, where different species or strains may reside compared to the other basins. Conversely, the database contains 173 sequences of *Dinobryon*, yet this number of reference sequences was not sufficient to allow the detection of this genera in the Skagerrak and Kattegat regions. In this study, the unique gradient of environmental conditions stresses the importance of genetic diversity at genus and species level, and the urgent need of taxonomist work to isolate and sequence taxa from diverse environments to complement reference databases in order to improve the taxonomical performance of metabarcoding methods.

The complementary nature of the two methods is further illustrated by species detected exclusively by one approach. Several taxa were recorded only by the Utermöhl method, likely because corresponding reference sequences are absent or insufficiently represented in the PR^2^ database. For example, *Peridiniella* (Dinophyceae), one of the most abundant genera in our dataset, was detected solely by microscopy (Fig. 4), and rare species such as *Gymnodinium stellatum*, *Thalassiosira eccentrica*, and *Tabellaria fenestrata* were likewise only identified microscopically. Conversely, metabarcoding exclusively detected genera such as *Choricystis* (Trebouxiophyceae), *Biecheleria* (Dinophyceae), *Haptolina* and *Prymnesium* (Coccolithophyceae), and *Pelagodinium* (Dinophyceae), which are small-celled taxa that are difficult or impossible to identify to genus level based on morphological characters alone under light microscopy. These findings underscore the importance of improving both the completeness and the reliability of public reference databases, where incorrectly annotated or unresolved sequences can lead to misassignment, while simultaneously maintaining morphological expertise that remains essential for taxa lacking molecular references.

The identification of small taxa (< 10 µm) using the Utermöhl method is known to be a challenging task^19^, and this difficulty is further amplified in diverse environments. For example, the Swedish west coast harbors a wide range of cryptomonads, making species identification based on morphology alone a difficult task^40^. As a result, these organisms are often reported as unknown cells belonging to the order Cryptomonadales. Our findings indicate that the application of metabarcoding techniques reveals the presence of genera like *Teleaulax*, which are frequently observed in the Baltic Sea, in the Skagerrak and Kattegat regions as well.

Similarly, the haptophyte genera *Chrysochromulina* and *Prymnesium*, known to include species responsible for some types of HABs^41^, are often identified only at the order level (Prymnesiales) in our microscopy dataset. Nevertheless, the application of metabarcoding has proven successful in detecting these genera in the Skagerrak, Kattegat, and the Baltic Sea. These findings emphasize the potential of metabarcoding as an early warning system for detecting HAB-forming taxa. Metabarcoding, with its ability to assign and annotate taxonomic units at a high level of detail using ASVs, enables us to differentiate community compositions between geographically and environmentally similar areas, such as the Skagerrak and Kattegat. This fine taxonomic discrimination of small-sized taxa allows for a precise assessment of the variations in community structure within these closely located regions.

Despite the higher number of species and genera detected by metabarcoding compared to microscopy, about the same number of rare species was found with both methods. Metabarcoding is considered to catch a higher diversity than microscopy^42^ and is therefore expected to detect a higher number of rare species. However, if the amount of DNA template is related to the DNA extracted and the volume filtered, then the equivalent volume of seawater analyzed by metabarcoding can be compared to the volume counted by microscopy. In this study, the seawater volume equivalent analyzed by metabarcoding was 1–23 ml, while the seawater volume counted with microscopy was 0.5–25 ml. The similar range of volumes analyzed by both methods would likely explain the similar number of rare species found by both methods in this study.

### Absolute quantifiability of metabarcoding results

Previous comparisons between metabarcoding and microscopy show inconsistency between the two methods depending on the phytoplankton group^11,14,16,18,21,22^. These discrepancies are likely related to some of the technical challenges that arise when quantifying community composition using metabarcoding. These include sample preservation^43^, DNA extraction^43,44^, primer choice^21,25^, PCR bias^35^, and reference database incompleteness^11^. Notably, variations in gene copy numbers per cell of the 18S small ribosomal subunit (SSU) across different taxa contribute the greatest uncertainty when estimating the contribution of specific taxa to the overall community^28,29^, which likely cause a discrepancy between abundance derived from metabarcoding and microscopic analyses^14^. Relative abundance data provided the best correlation between the two methods in our study (both on class and genus level) and was further enhanced by comparing relative read abundances to relative carbon concentration, in agreement with Andersson, et al.^22^. This adjustment increased the overall R^2^ value from 0.23 to 0.57 at the class level. Notably, this improvement was particularly pronounced for Cryptophyceae, Dinophyceae, and Coccolithophyceae (Fig. 6), possibly as taxa within these classes exhibited a stronger correlation between gene copy number and cell size rather than abundance, which is common in marine phytoplankton^12^. Attempts to improve normalization using DNA concentration or spike-DNA concentration did not substantially increase R^2^ values and generally performed worse than relative abundance normalization. Interestingly, the success of spike-normalization appeared to depend on the geographical area, potentially influenced by the lower phytoplankton biodiversity and extensive coverage of PR^2^ observed in freshwater environments^45^.

## Conclusion

Metabarcoding can serve as a valuable complement or addition to conventional microscopy analysis for long-term phytoplankton monitoring. Here, we have compared the two methods within a region distinguished by a pronounced salinity gradient spanning the Baltic Sea, Kattegat, and Skagerrak system, which hosts a diverse array of phytoplankton taxa. In comparison to the Utermöhl method, metabarcoding revealed a high diversity of eukaryotic micro- and nanophytoplankton and showed lower variability within samples. Nevertheless, challenges remain due to unreliable species-level annotation, which could be particularly problematic if 18S metabarcoding were applied to detect species responsible for HABs, as well as the semi-quantitative nature of the method, which limits accurate estimation of abundances and biomass critical for modeling and water quality assessment. Despite attempts at normalization, no method has been identified that can accurately infer absolute abundances from 18S rRNA gene sequence reads.

There is limited evidence to suggest that metabarcoding could replace microscopic analyses in long-term monitoring programs at present. Critically, improving the completeness and reliability of public reference databases remains an important priority, alongside the development of curated, quality-controlled reference libraries, particularly for taxa from underrepresented environments such as brackish waters. Continued progress in these areas will further strengthen metabarcoding as a valuable complement to conventional methods. As technology advances, including the adoption of long-read sequencing, and as more extensive work with normalization methods (spike-in or other), and estimation of gene copy numbers per cell is conducted, the accuracy of quantitative metabarcoding data will increase. Nonetheless, metabarcoding has the potential to serve as an effective complement to broaden biodiversity assessments in existing monitoring efforts.

## Methods

### Sampling program

In total, 232 samples were collected for phytoplankton analysis from January 2019 to February 2020 at 17 monitoring stations in the Baltic Sea and the adjacent Kattegat and Skagerrak during monthly/bimonthly sampling cruises (Fig. 1, Table 1). Samples were collected in connection with the Swedish National Marine Monitoring Program which also includes measurements of zooplankton, chlorophyll a, inorganic nutrients, salinity and temperature following standard methods^46^. A detailed description of the sampling procedure is presented in Latz, et al.^36^. Depth-integrated phytoplankton samples were collected using a tube between 0–10 m depth (0–20 m depth at two stations and 0–5 at one station, see Table 1). Samples for salinity were collected from discrete depths, analyzed using methods described by the Helsinki Commission (HELCOM)^7^ and averaged over 0–10 m.

In addition, samples were collected for technical validation of sample volume filtered for metabarcoding and method replicability. Different water volumes were tested for metabarcoding on three occasions at station Släggö on the Swedish Skagerrak coast in 2019 (on May 6^th^, August 6^th^ and October 7^th^) where five replicates of each volume of 10, 100, 200 and 500 ml were filtered. The intra-sample variability of the microscopy and metabarcoding methods were compared on three occasions in 2019 at station Släggö (on May 6^th^, August 6^th^ and October 7^th^) and three occasions at station B3/B7 in the northern Baltic Sea (on April 29^th^, June 25^th^ and August 20^th^), where five replicate samples were collected for each method.

### Microscopy-based phytoplankton analyses by the Utermöhl method

After collection, phytoplankton samples were immediately fixed in acidic Lugol’s iodine solution and stored in darkness until analysis. Micro- and nanophytoplankton composition, cell abundance, biovolume, and biomass were assessed by the Utermöhl sedimentation chamber method^2,3^, using a 25 ml sedimentation chamber. A detailed protocol can be accessed under Annex C-6 of the HELCOM-COMBINE manual^7^. Cell volume information originates from the HELCOM Expert Group on Phytoplankton^33^ updated in 2022 and supplemented with additional information for species in the Skagerrak. Conversion from cell volume to carbon biomass was based on equations described in Menden-Deuer and Lessard^47^.

### DNA metabarcoding of the 18S rRNA gene

The methods used for DNA sampling, extraction, amplification, sequencing and bioinformatics are described in detail in Andersson, et al.^44^ and Latz, et al.^36^. In brief, samples for DNA extraction were collected from the same depth-integrated water sample as the microscopy samples. Within an hour after sample collection, 200–500 ml of sea water was filtered onto mixed cellulose esters membranes with diameter of 47 mm and a pore size 0.22 µm (GSWP04700, Merck Millipore Ltd, Cork, Ireland). Filters were stored at − 20 °C immediately after sampling and transferred to − 80 °C within ten days after sampling. DNA extraction was performed using ZymoBIOMICS DNA Miniprep Kit (Zymo Research Corp, Irvine, CA, USA)^44^. After adding a lysis buffer to the filter, a known and consistent amount of synthetic spike-in DNA was added to each sample. The spike-in consisted of artificially designed DNA sequences that do not match any known organisms, allowing them to be unambiguously identified in the sequencing data. By comparing the number of spike-in sequence reads to the reads of natural taxa in each sample, an estimate of the absolute abundance of taxa sequences per volume of water can be derived, provided that the efficiency of DNA co-extraction and amplification is comparable between the spike-in and natural community DNA. The detailed design and validation of the spike-in sequences are described in Latz, et al.^36^.

The eukaryotic hypervariable region V4 of the 18S rRNA gene was amplified using the primer pair V4F (CCAGCASCYGCGGTAATTCC) and V4RB (ACTTTCGTTCTTGATYRR)^48^, complemented with 5’-end Illumina sequence adapters. DNA was amplified by using the polymerase chain reaction (PCR) protocol described in Latz, et al.^36^. The amplicons were purified using the MagSi-NGS PREP Plus Kit (MDKT00010075, magtivio BV., Nuth, the Netherlands) and amplified a second time with index-adapters. DNA libraries were multiplexed and sequenced by Illumina MiSeq (Illumina Inc, San Diego, CA, US) technology using a 3 × 300 bp configuration at the National Genomics Infrastructure, SciLifeLab, Stockholm, Sweden. Quality filtering, denoising and removal of potential chimeras of the demultiplexed sequences were performed using Cutadapt^49^ and DADA2^50^. Amplicon sequencing variants (ASVs) were classified with ‘assignTaxonomy’ in DADA2 using the Protist Ribosomal Reference (PR^2^) v5.1.0 database^23^. More details on how the data were processed are provided in Latz, et al.^36^.

### Data analysis

The eukaryotic phytoplankton identified in this study can be categorized into 14 micro- and nanophytoplankton classes that were consistently identified by both described methods, and are listed in Table 2. Some taxonomic modifications were applied to the taxa tables to better match each other and to follow the current AlgaeBase taxonomic database^37^. Lists of modifications are provided in Supplementary tables S1 and S2. The diatom classes Coscinodiscophyceae and Mediophyceae were combined for better method comparison, since unknown centric diatom cells identified by microscopy are often reported as unknown cells from the paraphyletic order Centrales. Organisms not identifiable to the class level by microscopy assigned to categories unicells or flagellates were excluded from the dataset.

DNA sequence read abundances were normalized to amount of spike-in DNA added per sample volume and spike sequence read counts, in an attempt to acquire absolute sequence abundance (number of molecules of each ASV per volume of water). In addition to spike-normalization, DNA-normalized read abundances were calculated by multiplying relative abundance (to the whole community, excluding spike-in reads) by DNA concentration in the sample after extraction. Proportions of 18S rRNA sequence reads, cell abundance, biovolume, and carbon biomass concentration were calculated relative to the defined eukaryotic phytoplankton community, i.e. the sum of all measured values within the classes listed in Table 2. The proportion of sequence reads that were assigned to these classes relative to all reads (excluding spike-in reads) are listed in Table 1.

Linear regressions were carried out between each metabarcoding metric (raw reads, spike-normalized counts, or DNA-normalized counts) and the corresponding microscopy measurements (cell abundance, carbon concentration, biovolume concentration) to evaluate the effectiveness of the normalization techniques. The dataset had been aggregated to class level before analysis, and each data point in the regression model represents the value for one class in one sample.

The software R^51^ was used for processing data, including statistical analyses to compare 18S rRNA gene metabarcoding to the Utermöhl method. Components of ‘tidyverse’ package^52^ were used for data management and visualization. The R library ‘ggOceanMaps’ was used for producing maps^53^, the ‘phyloseq’ and ‘MicrobiotaProcess’ packages for managing and comparing microbiome datasets^54,55^, and the ‘vegan’ package was used for permutational multivariate analysis of dispersion (PERMDISP) and permutational analysis of variance (PERMANOVA) analysis using 999 permutations^56^.

## Supplementary Information

Supplementary Information.

## Data Availability

DNA sequences have been deposited to the European Nucleotide Archive under the accession number https://identifiers.org/ena.embl:PRJEB55296. All data presented in this study are described in detail in Latz, et al.^36^ and can be accessed through the SciLifeLab Data Repository at 10.17044/scilifelab.20751373.v1 ref^57^. All R code that was used for post-processing of data are available on Zenodo at 10.5281/zenodo.17977672.
