# Supplementary material for: Comparing DNA metabarcoding with light microscopy to identify eukaryotic phytoplankton in the Baltic Sea, Kattegat and Skagerrak
Source: Sci Rep. 2026 May 19;16:15743. doi: 10.1038/s41598-026-48838-z (PMC13190673; doi:10.1038/s41598-026-48838-z)
Supplement: Supplementary file 1 — Supplementary Information. [file 41598_2026_48838_MOESM1_ESM.pdf]

Supplementary Table S1. List of taxonomic corrections applied to the taxa identified by the Utermöhl method to match the Protist Ribosomal Reference (PR^2) database release (5.1.0.) and the current AlgaeBase taxonomical database [34].

Original name	Translated name	Taxonomic rank
Coscinodiscophyceae	Coscinodiscophyceae + Mediophyceae	Class
Mediophyceae	Coscinodiscophyceae + Mediophyceae	Class
Noctilucea	Noctilucophyceae	Class
Actinocyclus octonarius var. *octonarius*	*Actinocyclus octonarius*	Species
Amylax triacantha var. triacantha	*Amylax triacantha*	Species
Ceratium horridum	*Tripos longipes*	Species
Chaetoceros ceratosporus var. *ceratosporus*	*Chaetoceros ceratosporus*	Species
Chaetoceros gracilis	*Chaetoceros neogracilis*	Species
Chaetoceros subtilis var. *subtilis*	*Chaetoceros subtilis*	Species
Chaetoceros throndsenii var. *throndsenii*	*Chaetoceros throndsenii* var. *throndsenia*	Species
Desmodesmus armatus var. *armatus*	*Desmodesmus armatus*	Species
Dictyocha speculum	*Octactis speculum*	Species
Emiliania huxleyi	*Gephyrocapsa huxleyi*	Species
Fragilaria capucina var. capucina	*Fragilaria capucina*	Species
Goniochloris smithii	*Pseudogoniochloris tripus*	Species
Gonyaulax verior	*Sourniaea diacantha*	Species
Gymnodinium simplex	*Protodinium simplex*	Species
Gymnodinium vestificii	*Kapelodinium vestifici*	Species
Heterocapsa triquetra	*Kryptoperidinium triquetrum*	Species
Kathablepharis remigera	*Katablepharis remigera*	Species
Katodinium glaucum	*Lebouridinium glaucum*	Species
Lingulodinium polyedra	*Lingulaulax polyedra*	Species
Monoraphidium arcuatum	*Ankistrodesmus arcuatus*	Species
Odontella sinensis	*Odontella chinensis*	Species
Plagioselmis	*Teleaulax*	Genus
Plagioselmis prolonga	*Teleaulax amphioxeia*	Species
Pleurochrysis	*Chrysotila*	Genus

Pleurochrysis carterae	*Chrysotila carterae*	Species
Prorocentrum compressum	*Tryblionella compressa*	Species
Rhizosolenia setigera	*Sundstroemia setigera*	Species
Rhizosolenia setigera f. *pungens*	*Sundstroemia setigera*	Species
Synedra ulna	*Ulnaria ulna*	Species
TetraÃ«dron minimum	*Tetraedron minimum*	Species
Tetraëdron minimum	*Tetraedron minimum*	Species
Tripos horridus	*Tripos longipes*	Species

Supplementary Table S2. List of taxonomic corrections applied to the taxa identified by 18S rRNA gene metabarcoding by annotation by the Protist Ribosomal Reference (PR^2) database (release 5.1.0.), to match the current AlgaeBase taxonomical database [34] and the microscopy dataset.

Original name	Translated name	Taxonomic rank
Coscinodiscophyceae	Coscinodiscophyceae + Mediophyceae	Class
Mediophyceae	Coscinodiscophyceae + Mediophyceae	Class
Prymnesiophyceae	Coccolithophyceae	Class
Synuraceae	Mallomonadaceae	Family
Melkoniana	*Melkoniania*	Genus
Actinoptychus octonarius	*Actinocyclus octonarius*	Species
Amphora semperpalorum	*Halamphora semperpalorum*	Species
Ataxiodinium choane	*Ataxiodinium choanum*	Species
Bacillaria paxillifer	*Bacillaria paxillifera*	Species
Chaetoceros brevis 3	*Chaetoceros brevis*	Species
Chaetoceros cf tortissimus	*Chaetoceros tortissimus*	Species
Chaetoceros curvisetus 1	*Chaetoceros curvisetus*	Species
Chaetoceros debilis 1	*Chaetoceros debilis*	Species
Chaetoceros diadema 1	*Chaetoceros diadema*	Species
Chaetoceros didymus 2	*Chaetoceros didymus*	Species
Chaetoceros gracilis	*Chaetoceros neogracilis*	Species
Chaetoceros peruvianus 1	*Chaetoceros peruvianus*	Species
Chaetoceros pumilum	*Chaetoceros calcitrans f. pumilus*	Species
Chloroidium saccharophila	*Chloroidium saccharophilum*	Species
Choricystis minor	*Choricystis parasitica*	Species
Cyclotella meneghiniana	*Stephanocyclus meneghinianus*	Species
Diatoma tenue	*Diatoma tenuis*	Species
Dictyocha globosa	*Vicicitus globosus*	Species
Dictyocha speculum	*Octactis speculum*	Species
Dictyochloropsis symbiontica	*Symbiochloris symbiontica*	Species

Fragilidium duplocampanaeforme	*Fragilidium duplocampaniforme*	Species
Gonyaulax digitale	*Gonyaulax digitalis*	Species
Gonyaulax elongata	*Gonyaulax ovum*	Species
Heterocapsa nei/rotundata	*Heterocapsa niei/rotundata*	Species
Heterochlorella luteoviridis	*Jaagicholrella luteoviridis*	Species
Kryptoperidinium foliaceum	*Kryptoperidinium triquetrum*	Species
Lingulodinium polyedra	*Lingulaulax polyedra*	Species
Minidiscus comicus	*Mediolabrus comicus*	Species
Monodus subterranea	*Monodopsis subterranea*	Species
Mychonastes zofingiensis	*Chromochloris zofingiensis*	Species
Pelagodinium beii	*Pelagodinium bei*	Species
Physomonas elongata	*Pedospumella elongata*	Species
Plagioselmis prolonga	*Teleaulax amphioxeia*	Species
Prasinopapilla vacuolata	*Pyramimonas vacuolata*	Species
Pseudopedinella elastica	*Pedinella elastica*	Species
Spumella elongata	*Physomonas elongata*	Species
Synedra fragilaroides	*Synedra fragilarioides*	Species
Rhizosolenia delicatula	*Guinardia delicatula*	Species
Rhizosolenia robusta	*Neocalyptrella robusta*	Species
Rhizosolenia setigera	*Sundstroemia setigera*	Species
Scenedesmus abundans	*Desmodesmus abundans*	Species
Scenedesmus armatus	*Desmodesmus armatus*	Species
Scenedesmus obliquus	*Tetradesmus obliquus*	Species
Synedra fulgens	*Synedrosphenia fulgens*	Species
Thalassiosira concaviuscula	*Thalassiosira aestivalis*	Species
Thalassiosira guillardii	*Conticribra guillardii*	Species

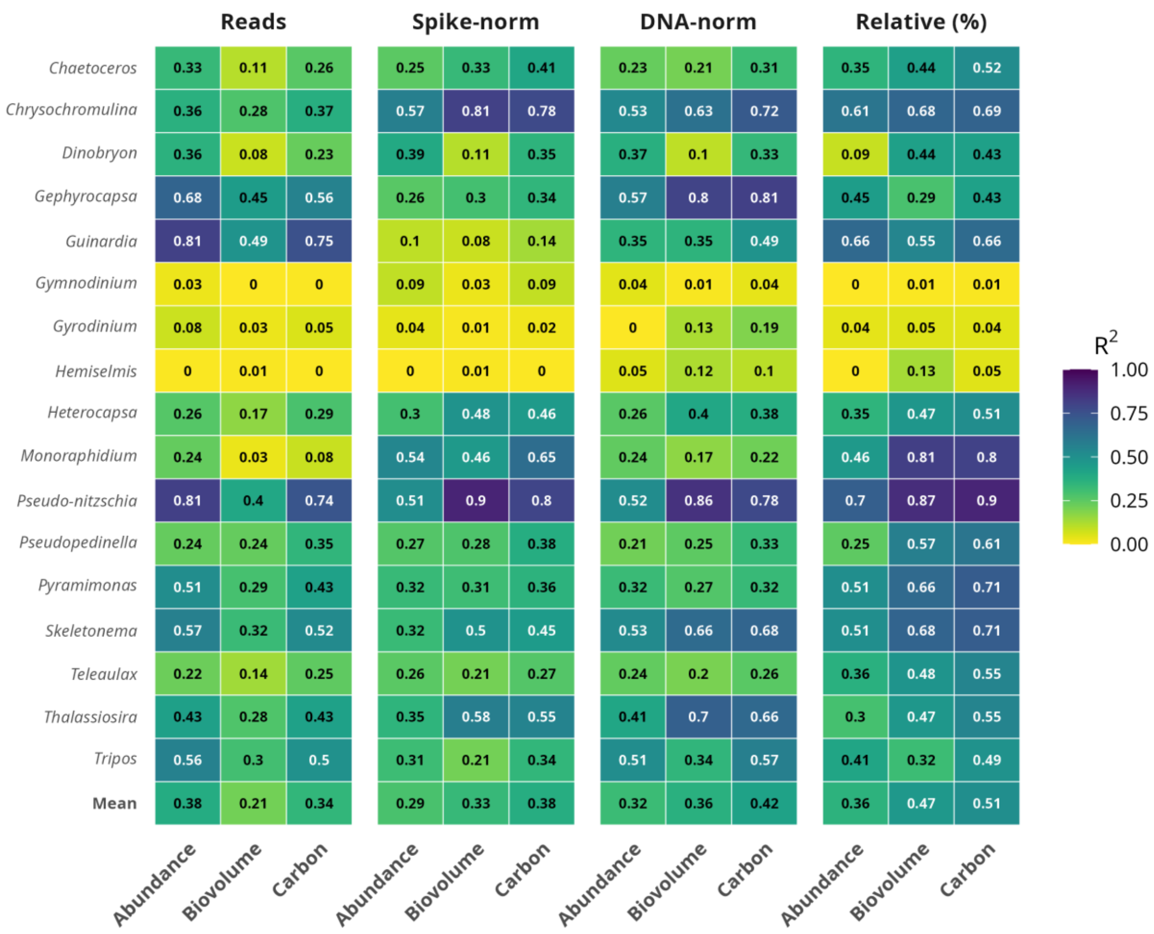

Supplementary Figure S3. Heatmap of R^2 values between microscopy and metabarcoding genus-level abundances for the 17 most abundant genera in the dataset. Values are calculated on log-transformed microscopy and metabarcoding data (raw reads, spike-normalized, DNA-normalized, and relative abundances) compared with cell abundance, carbon, and biovolume concentrations. Each tile shows the R^2 (rounded to two decimals) for a genus–group pair, with color intensity indicating the strength of the relationship.
